# Introduction of a New Interesting Walnut Cultivar “Leto”

**DOI:** 10.3390/plants10122738

**Published:** 2021-12-13

**Authors:** Ioannis Manthos, Dimos Rouskas

**Affiliations:** Department of Nut Trees, Institute of Plant Breeding and Genetic Resources, Hellenic Agricultural Organization-Dimitra, Neo Krikelo, 35100 Lamia, Greece; dimos.rouskas@yahoo.com

**Keywords:** walnut, *Juglans regia*, Greece, phenology, lateral bearing, walnut description

## Abstract

Ιn an effort to create walnut cultivars (*Juglans regia*) with high productivity, fruit quality and lateral bearing, a new cultivar, named “Leto”, was created by the cross “Gustine” × “Pedro”. Its main phenological and pomological characteristics were assessed according to the criteria of IPGR (1994) and UPOV-TG/125/6 (1999), for 10 consecutive years and compared with its maternal cultivars and “Chandler”. Observations showed that “Leto” has high lateral bearing habit (90%) and presents satisfactory yield at the full production age. The tree size is smaller than that of its parents and “Chandler”, female flowers bloom from 11 to 22 of April and male from 3 to 6 of April. “Leto” nuts are harvested at the end of September, present easy hull dehiscence and high kernel percentage. Other positive nut characteristics of “Leto” are light kernel color, well kernel filling and easy removal of the kernel halves. “Leto” is a mid-early cultivar of great interest due to its high- quality nuts, suitable for dense plantings, in regions where the last spring frosts occur in late March to early April, thus, making it a promising cultivar for Greece, but also for other regions with similar geomorphological and climatic conditions.

## 1. Introduction

The Persian walnut (*Juglans regia* L.) is one of the most economically important cultivated nut tree species. Walnut tree cultivation is widely distributed and, on a global basis, walnuts rank first in nut production (4,498,442 Mt), following by cashews and almonds [1]. During the last decade, worldwide walnut production has doubled, due to the recognition of its beneficial effects for consumers’ health. Various studies and reviews have highlighted the importance of walnut consumption as part of a regular, healthy diet, due to their content, including a wide range of nutrients and many phytochemical species that may be important to human health, such as a-linolenic acid and other PUFAs, phytosterols, minerals, γ-tocopherol, polyphenols and others, that exhibit antioxidant properties and offer protection against the development of many cardio-vascular related diseases, age-related neurological disorders, incidence of diabetes, and even some types of cancer [2,3,4,5,6]. 

In Greece, walnut production ranks first among all nuts. With an average production of 31,040 tons (year 2019) [1], Greece is the third largest walnut producer in the European Union after Romania and France. Despite the remarkable production of nuts, Greece imports nuts, mainly walnut kernels (4071 tons in 2019) [1].

Walnut cultivation in Greece is characterized by two completely different situations. On the one hand, there are the new walnut orchards, which are increasing progressively, in which selected universal cultivars are cultivated, such as the lateral bearing cultivars, “Chandler”, “Lara” and “Pedro”, in the semi-mountainous and lowland areas, and the apical bearing cultivars, Franquette and Ronde de Montignac, in the mountainous and high-mountainous regions. On the other hand, there are the traditional, old walnut populations (seedlings), the cultivation of which has decreased over the years. Twenty-seven and seventy of these seedlings have been previously evaluated by Rouskas et al. (1997) [7] and Rouskas and Zakynthinos (2001) [8], respectively. Of the seedlings evaluated, a large number showed lateral bearing habit, and the majority were characterized as early-breaking material. According to the study authors, no genetic material was found that could be linked directly to the “great cultivar”, but there was very valuable genetic material that could be used as genitors in pre-planned crosses directed towards the genetic improvement of walnuts.

Nut production is progressively moving from a traditional situation to a new one involving an intensive approach which emphasises quality of the plant material (e.g., cultivars with lateral fruiting and rootstocks), increased plantation tree density, and mechanization of cultivation, harvesting and post-harvesting, which are some the features that are very important for the development of the Greek walnut industry today [8]. Furthermore, the relief of the Greek mountains influences the climatic conditions and creates a variety of suitable regions for walnut cultivation [9]. Therefore, there is a great need to develop new and improved walnut cultivars that best meet the needs of the growers, as well as the specific characteristics of the respective growing areas. This need worldwide, together with the fact that the walnut industry is faced with substantial losses due to pests and diseases affecting both cultivars and rootstocks, has driven the development of walnut breeding programs in the major producing countries. Many breeding programs for walnut varieties have been carried out in the USA, Turkey, Iran, France, and Hungary; some of these programs are still running [10,11,12,13,14,15]. The main goals of breeding in walnut production are high yield and kernel quality. Other characteristics, such as lateral bearing, nut and kernel weight, nut size, kernel percentage, shell thickness, kernel color and ease of kernel removal, are among the important objectives of walnut breeding, along with tolerance to biotic and abiotic factors [7,12,13,14,16,17,18]. As a result of global warming and climate change, some other breeding objectives have also been considered, such as late leafing to avoid spring late frost, which is one of the most serious problems causing loss of production in walnuts. Late leafing is a desirable attribute for walnuts grown in regions prone to late spring frosts [19]. 

Efforts have been made by the Department of Nut Trees of Hellenic Agricultural Organization (HAO)—DIMITRA, from 1985 to the present day, to create new distinctive walnut cultivars, with high productivity and good fruit quality, suitable for the soil and climatic conditions in different regions of Greece, and resistant to pests and diseases, as well as to develop cultivars with late leafing, high lateral fruit bearing, and early harvest dates. Some of the grafted seedlings that have been evaluated [7,8] were selected because of their desirable nut traits and their resistance to walnut blight and anthracnose, as well as for their lateral bearing habit, etc. Their crosses with commercial cultivars (e.g., “Chandler”, “Gustine”, “Franquette”) have been established in the department’s collections and are now being assessed. In addition, crosses between well-known commercial cultivars (e.g., “Chandler”, “Hartley”, “Gustine”, “Pedro”, “Franquette”) have been performed to take advantage of each cultivar’s pertinent traits. Recently, through specific crosses, a lateral bearing cultivar with satisfactory traits, called “Ourania”, has been developed. It is a new walnut cultivar of Greek origin that was created by the cross between “Hartley” and “Gustine”. It is a mid-late cultivar, with similar pomological characteristics to “Hartley” but has a high lateral bearing habit. It is a walnut cultivar of great importance, not only for the region of Greece, but also for other countries, suitable for cultivation in semi-mountainous and mountainous regions, where the last spring frost occurs until mid-April [20]. At the same time, from another cross between “Gustine” and “Pedro”, a new cultivar was created, named “Leto”. “Gustine” was selected as the maternal genetic material mainly because of its high lateral bearing habit and light kernel color, whereas “Pedro” was chosen for the good hull dehiscence and for the later bud break compared to “Gustine”. Thus, the aim of the present study was to provide insights into the characteristics of the newly created “Leto” cultivar and its performance. Comparisons of the most important phenological and pomological characteristics were made with its parental cultivars “Gustine” and “Pedro”, as well as with the best-selling walnut cultivar “Chandler”.

## 2. Results

### 2.1. Tree and Phenological Characteristics 

According to the characteristics observed from 2010 to 2019, shown in Table 1, “Leto” was shown to be a protandrous cultivar like other cultivars studied. Its trees had low vigor, a spreading habit, and dense branching, similar to the parental cultivars. Additionally, “Leto’s” lateral bearing habit was found to be 90%, as high as “Chandler”, similar to its maternal cultivar “Gustine”, but higher than its paternal cultivar “Pedro” that showed an 80% lateral bearing habit. The first catkins appeared in the 3rd year of age, while the first female flowers appeared in the first year of age. For the year studied, the level of male flower abundance in “Leto” was characterized as intermediate, comparable to other cultivars, while it exhibited easy hull dehiscence, similar to the paternal cultivar “Pedro” and “Chandler”, and in contrast to “Gustine”, which had lower hull dehiscence (Table 1).

“Leto’s” mean in shell nut production was 29.37 ± 4.05 kg with no difference from its parental cultivars or “Chandler”. With respect to tree size parameters, the radius of the crown of “Leto” was significantly smaller than that of “Gustine” and “Chandler” (*p* < 0.05), and the trunk circumference was smaller than for all three comparison cultivars (*p* < 0.05) (Table 2).

**Figure 1:**
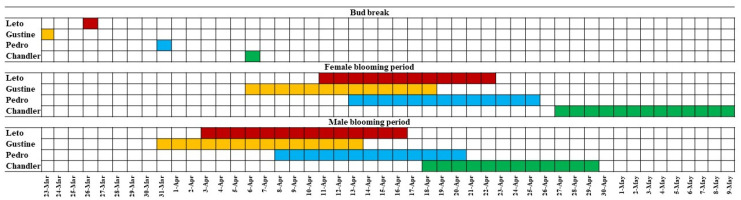
Mean phenological characteristics (observation for 10 consecutive years) of “Leto” walnut cultivar compared to its parental cultivars “Gustine” and “Pedro” and “Chandler”, concerning date of bud break, first and last female and male bloom dates.

The mean values of the phenological characteristics of walnut cultivars during the observations for ten consecutive years are given in Table 3. Bud break of “Leto” occurred on 26 March ± 6.61 days, close (*p* < 0.05) to its maternal cultivar “Gustine” (23 March ± 6.60 days), 5.3 days earlier than its paternal cultivar “Pedro” (31 March ± 5.16 days), and 11.6 days earlier than “Chandler” (6 April ± 8.03 days), (*p* < 0.001). The beginning and end of female blooming of “Leto” occurred at 11 April ± 5.84 days and at 22 April ± 6.11 days, respectively, similar to the “Pedro” cultivar. First female blooming of “Leto” occurred 4.1 days later than “Gustine” and 16.4 days earlier than “Chandler” cultivar (*p* < 0.001), and last female blooming occurred 4.1 days later than “Gustine” and 15.9 days earlier than “Chandler” (*p* < 0.001). The beginning and end of male blooming of “Leto” occurred at 3 April ± 6.57 days and at 16 April ± 5.95 days, respectively, similar to its maternal “Gustine” cultivar. First male blooming of “Leto” occurred 4.4 days earlier than “Pedro” and 14.4 days earlier than “Chandler” cultivar (*p* < 0.001), and last male blooming occurred 3.8 days earlier than “Pedro” and 12.2 days earlier than “Chandler” (*p* < 0.001) (Table 3; Figure 1). Harvest of nuts of “Leto” occurred at 22 September ± 3.10 days, close to “Pedro”, 2.9 days later than “Gustine” and 10.2 days earlier than “Chandler” (*p* < 0.001) (Table 3). The overlap percentage was higher (*p* < 0.001) between “Leto” and parental cultivars compared to “Chandler” (Table 3).

Concerning biotic and abiotic factors, “Leto” presented intermediate sunburn susceptibility of the hull, in contrast to its parental cultivars that presented high sunburn susceptibility, intermediate susceptibility to codling moth (*Cydia pomonella* L.), and lower (intermediate) susceptibility to walnut blight (*Χanthomonas arboricola* pv. *juglandis*) than “Gustine” (Table 4). 

### 2.2. Nuts Characteristics

**Figure 2:**
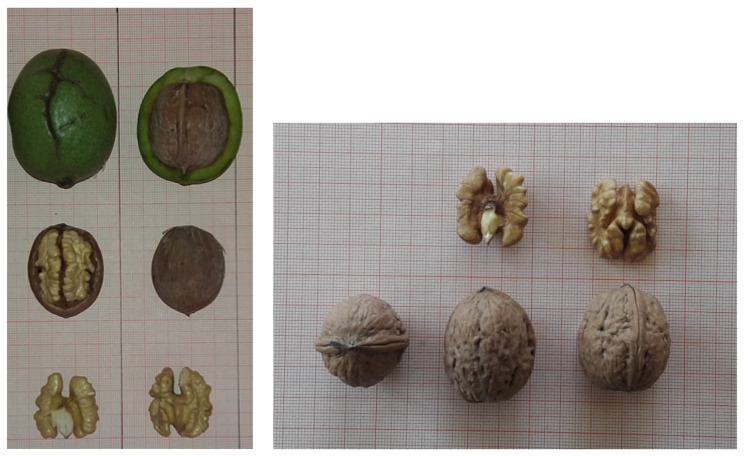
Nut and kernels of “Leto” cultivar.

Fruit characteristics are presented in Table 5 and Table 6 and were collected over 10 consecutive years. Measurements concerning nut dimensions and weights are presented as mean ± standard deviation (SD). “Leto” nuts were of medium size and of ovate shape, similar to nuts of “Gustine” (Table 5; Figure 2). Nut height, nut width, nut thickness and roundness of “Leto” cultivar differed significantly (*p* < 0.001; *p* < 0.01 and *p* < 0.001, respectively) from that of “Pedro” and “Chandler”, but not from “Gustine” cultivar. The shell was evaluated to be of medium texture, and light color, with complete integrity, similar to the “Gustine” cultivar (Table 3; Figure 2). Shell thickness of “Leto” did not differ significantly from “Gustine” and “Pedro”, but “Chandler” nut shells were found to be thinner (*p* < 0.01) in comparison to the three other cultivars (Table 6). 

“Leto” nut shape in longitudinal section through suture was evaluated as ovate (similar to parental cultivars) and in longitudinal section perpendicular to suture as trapezium (different to parental cultivars). The shape in cross section was evaluated as oblate (like “Pedro”), and the shape of base perpendicular to suture as rounded, similar to both parental cultivars. The shape of apex perpendicular to suture was rounded (different to parental cultivars) and the prominence of the apical tip was found to be medium (similar to parental cultivars). The position of pad on suture was found on the upper 2/3 of the nut, the prominence of pad on suture was medium (similar to parental cultivars), the width of pad was narrow and the depth of groove along pad on suture was medium (similar to its maternal cultivar “Gustine”). The structure of the surface of “Leto” nut shell was slightly grooved and shell thickness was thin. Nuts presented medium adherence of the two halves of shell and thin thickness of the primary and secondary membranes. All the above nut characteristics were equivalent to that of its maternal cultivar “Gustine” (Table 5; Figure 2).

The mean dried in shell nut weight of “Leto” cultivar was 12.96 ± 0.61 g, and did not differ significantly (*p* > 0.05) from that of its parental cultivars. However, “Leto” and “Pedro”, in shell nut weights were significantly lower than for “Chandler” (*p* < 0.01); “Gustine” did not differ significantly from “Chandler". The mean kernel weight of “Leto” nuts was 6.40 ± 0.54 g, which did not differ significantly either from its maternal cultivar, or from the reference cultivar “Chandler”. It is of interest that the kernel efficiency of “Leto” nuts was found to be significantly higher (*p* < 0.05) than for its paternal cultivar “Pedro”, numerically higher than its maternal cultivar “Gustine”, and similar to “Chandler” nuts (Table 6). “Leto” nuts presented light kernel color, with well kernel fill, and easy removal of the kernel halves, like its maternal cultivar “Gustine” (Table 5; Figure 2). 

## 3. Discussion

In the Department of Nut Trees of HAO—DIMITRA, to create new walnut cultivars suitable for the various geographical and meteorological conditions of Greece, but also for other Mediterranean and Mediterranean-like areas, characteristics such as late leafing, high lateral fruit bearing, early harvest dates, resistance to pests and diseases, high yield, and good nut quality, were considered when deciding on crosses. Selected seedlings, and their crosses with commercial cultivars (e.g., “Chandler”, “Gustine”, “Franquette”), as well as crosses between widespread cultivated cultivars (e.g., “Chandler”, “Hartley”, “Gustine”, “Pedro”, “Franquette”), have been established in collections in the department and are now being evaluated. So far, a lateral bearing cultivar called “Ourania”, which emerged from a cross between “Hartley” and “Gustine”, has been described and suggested for cultivation in semi-mountainous and mountainous areas [20]. In addition, the new “Leto” cultivar was created from the cross of “Gustine”, which was selected because of its high lateral bearing habit and light kernel color, and “Pedro”, which was selected because of good hull dehiscence and later bud break in relation to “Gustine”.

“Leto’s” phenological description shows that this new walnut cultivar has high lateral bearing habit, similar to “Chandler”, as well as the newly described “Ourania” [20]. Lateral bud fruitfulness is a significant characteristic and criterion when choosing a cultivar for plantation and one of the most important traits in walnut breeding programs [14,21], as this habit makes a genotype more productive in terms of yield capacity [22]. According to Hendricks et al. (1998) [23], varieties with high (80–90%) lateral bud fruitfulness bear more heavily in the early years than varieties with low (0–10%) lateral bearing-habit. “Leto” presents low tree vigor, is a protandrous cultivar, and flowers at early age, as female flowers appear in the first year, while catkins appear in the third year of age, similar to parental cultivars. It is a mid-early cultivar and it has an earlier bud break in relation to its paternal “Pedro”, as well as to “Chandler” and “Ourania”. According to Hendricks et al. (1998) [23], date of leafing and blooming are important considerations when choosing a cultivar. Early-leafing cultivars are susceptible to late spring frosts, thus reducing tree fruiting and yield, or they are susceptible to rain-related disease problems, such as walnut blight. For example, very early cultivars, such as “Serr”, could be cultivated in regions with a mild Mediterranean climate, while late cultivars, such as “Franquette”, could be cultivated in mountainous areas, where the last frosts often occur in late April. According to climatic diversity and the late spring frosts in Greece, four regions for walnut cultivation can be distinguished. The first includes the areas where the last spring frosts occur in the first fifteen days of March. The second region includes areas where the last spring frosts take place in late March. The third and fourth regions include areas where the last spring frosts occur in the first fifteen days of April and late April, respectively [8]. Thus, the newly created cultivar “Leto” is suitable for cultivation in regions where the last spring frosts occur in late March to early April, while “Ourania” is a mid-late cultivar and is suggested for cultivation in semi-mountainous and mountainous regions, where the last spring frosts occur until mid-April [20]. “Leto” female blooming starts and ends later than its maternal “Gustine” and earlier than “Chandler”, whereas male blooming starts and ends earlier than its paternal cultivar “Pedro”, and “Chandler”. 

Regarding tree size, “Leto” has a lower crown radius and trunk circumference compared to its parental cultivars and “Chandler”, a trait that make this new cultivar suitable for dense plantations as well as in unfavorable areas where mechanical harvesting is not possible.

“Leto” nut harvest occurs in late September, similar to “Pedro”, later than “Gustine”, but earlier than “Chandler” and “Ourania”. The harvest date is another important phenological parameter that determines the economic potential of a cultivar, which directly affects the spot value of the marketed products. Although this applies to walnut, an early harvest date can also be viewed as a protective measure against early-autumn frost events [24]. Early harvest dates offer walnut producers the opportunity to enter the walnut market earlier and thus achieve higher market prices. Another, benefit of an early harvest date is the reduced need for a walnut drying process, which saves energy and reduces drying costs. Therefore, the early harvest date of “Leto” can be considered another promising feature of the phenological traits of this cultivar.

“Leto” walnuts show easy hull dehiscence, similar to “Chandler”, intermediate sunburn susceptibility of the hull, in contrast to its parental cultivars which presented high sunburn susceptibility, intermediate susceptibility to codling moth, and lower susceptibility to walnut blight than “Gustine”. However, the “Ourania” cultivar presented lower sunburn susceptibility of the hull and lower susceptibility to walnut blight [20].

Pomological assessment helps to determine fruit quality and has therefore been widely used in many breeding studies to identify fruit cultivars with superior characteristics. According to the current results, the nuts of the “Leto” cultivar are medium in size and have an ovate shape and their dimensions, shell texture, color, and integrity, are similar to those of the maternal cultivar “Gustine”. Similar to the parental cultivar, the shell is thicker than “Chandler”. The most important characteristics when considering a walnut cultivar are the nut weight, the kernel weight, the kernel percentage, the kernel color, the kernel fill, and the easy removal of the kernel halves [24]. The mean dried nut weight (in shell) of “Leto” is similar to its parental cultivars, but lower to both “Chandler” and “Ourania” [20]. However, the mean kernel weight is high, similar to the reference cultivar “Chandler”. The latter leads to a high kernel percentage of “Leto” nuts (49.37 ± 2.81%), similar to “Chandler”, and higher than “Pedro”. “Ourania” showed a lower kernel percentage (46.92 ± 0.82%) [20]. The kernel percentage is considered a significant trait for the evaluation of a cultivar with regard to its productivity [23]. Other positive, desirable nut characteristics of “Leto” are its light kernel color, well kernel filling, and easy removal of the kernel halves. 

## 4. Materials and Methods

### 4.1. Materials and Experimentation Site Description

The study was carried out in the Department of Nut Trees of the Institute of Plant Breeding and Genetic Resources of HAO-DIMITRA. In an effort to obtain new productive and high-quality walnut genotypes, a cross between “Gustine” (maternal parent) and “Pedro” (paternal parent) by researcher Dimos Rouskas was made in 1986 as part of a European program (Noix UE, 1986). The certified parental cultivars were obtained from the Institut National de la Recherche Agronomique (INRA, Bordeaux). “Gustine” was chosen mainly for its high lateral bearing habit and light kernel color, while “Pedro” was chosen for its good hull dehiscence and the fact that the bud break occurs a week later than “Gustine”. The genotype was grafted onto seedling rootstock of the Lozeronne cultivar and the plants were planted in 1990 at a planting distance of 9 × 9 m. Collections of the parental cultivars “Gustine” and “Pedro”, as well as of “Chandler”, of similar age, were established in the same experimental orchard of the Department of Nut Trees and were included in the trial as reference cultivars.

The study area was located in Lamia, Fthiotida of Greece at the coordinates 38°49′36″ N 22°26′27″ E and at an elevation of 15 m and a slope of approximately 1%. In general, the same standard cultivation practices (e.g., fertilization, irrigation, pruning, pest and disease control) and care were applied to all trees of all cultivars studied. Irrigation was carried out through pipes with four micro drippers of 30 L/h around each tree. The applied pruning system was cup-shaped with a central axis for all trees of all cultivars examined.

Soil examination (at 0–30 cm, 30–60 cm, and 60–90 cm depth) provided the following characteristics: (a) mechanical soil composition: clay 52–56%, silt 32–36%, sand 8–14%, soil characterization as clayey [25], (b) water saturation 65–70%, electrical conductivity 0.55–0.59 mS/cm, total salts 0.2%, pH 7.7 [26], (c) calcium carbonate 11.0% and 2.8% organic matter.

### 4.2. Meteorological Data

**Figure 3:**
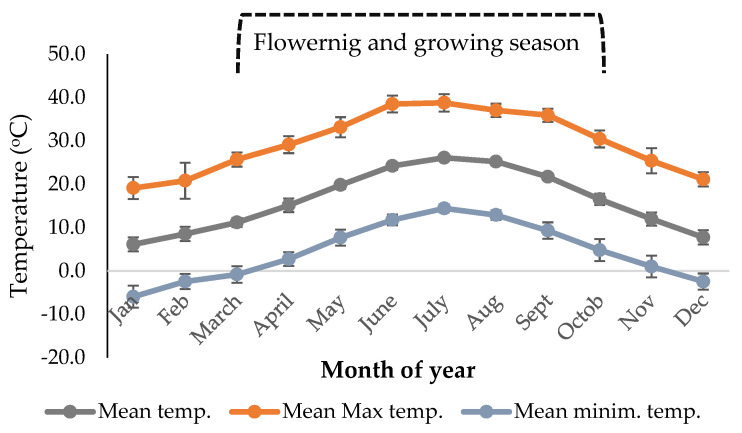
Mean temperature (°C), mean minimum and maximum temperature (°C), per month, for the years studied (2010–2019).

**Figure 4:**
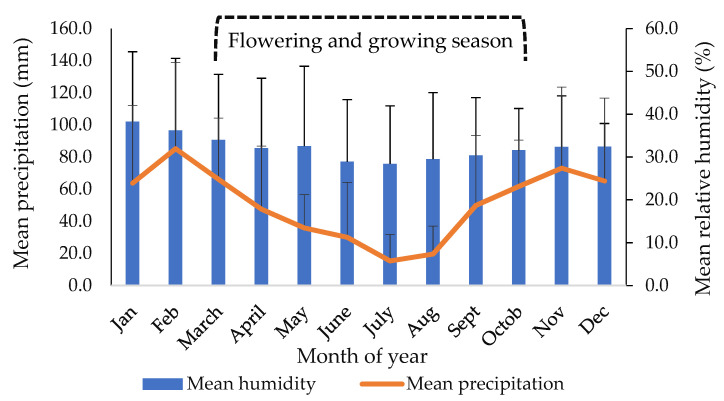
Mean precipitation (mm) and mean relative humidity (%), per month, for the years studied (2010–2019).

The meteorological data were collected from the meteorological station, which is set up in the institute’s facilities. For the years examined (2010–2019), meteorological data (e.g., average monthly temperature, minimum and maximum temperature, average monthly precipitation and relative humidity), for the studied years (2010–2019), were recorded and mean values (±SD) were calculated, as shown in Figure 3 and Figure 4.

### 4.3. Tree and Phenological Characteristics Observation 

Tree and phenological characteristics (Table 1, Table 2, Table 3 and Table 4) of the new cultivar “Leto” as well as its parental cultivars and “Chandler”, which were included in the trial, were collected, from ten (10) trees per cultivar, for ten consecutive years from 2010 to 2019, and were evaluated according to International Plant Genetic Resources Institute (IPGR, 1994) [27] criteria. 

### 4.4. Nut Traits Measurements and Observation

For the evaluation of the pomological characteristics (nut characteristics) of “Leto” and the other three reference cultivars (Table 5 and Table 6), 20 nuts per cultivar were collected at harvest time, each year, between 2010 and 2019 and assessed according to the criteria of IPGR (1994) [27] and UPOV-TG/125/6 (1999) [28]. Nut height, nut width, nut thickness and nut roundness index ((nut width + nut thickness)/2) × ut height) were defined according to the criteria of UPOV (1999) [28] and the measurements were carried out with a digital caliper of 0.01 mm. In addition, in shell nut weight and kernel weight were determined with a digital scale (0.01 g). The kernel weight to in shell nut weight ratio was calculated as kernel percentage (%). The nut shape and shell characteristics of the examined cultivars were determined according to the criteria of IPGR (1994) [27], as well as UPOV (1999) [28], as indicated in the tables. The color of the kernel was evaluated according to the criteria of UPOV (1999) [28].

### 4.5. Assessment of Susceptibility to BIOTIC and Abiotic Factors

The susceptibility of hulls of cultivars to sunburn was studied for two years (2018–2019) under natural conditions on summer days at temperatures above 42 °C. Nuts that were not covered by tree foliage and exposed to sunlight were examined for sunburn symptoms, and the degree of susceptibility was calculated (%). In order to categorize the sunburn sensitivity of each cultivar according to IPGR (1994), the following categorization was carried out: susceptibility scale 1: 0–20%, 3: 21–40%, 5: 41–60%, 7: 61–80% and 9: 91–100%. The susceptibility of nuts of cultivars to codling moth (*Cydia pomonella* L.) and walnut blight (*Χanthomonas arboricola* pv. *juglandis*) was investigated for two years (2018–2019). The susceptibility to both factors was assessed under usual phytosanitary orchard control. At the time of harvest, 100 nuts were randomly selected from each cultivar and the number of infected nuts was determined and the percentage (%) was calculated. In order to categorize the degree of susceptibility of each cultivar to codling moth and walnut blight according to IPGR (1994), the following categorization was made: susceptibility scale 0: no signs of susceptibility, 1: 0–20%, 3: 21–40%, 5: 41–60%, 7: 61–80% and 9: 91–100%. 

### 4.6. Statistical Analysis

To compare the bud break and flowering dates of the examined cultivars, calendar days were used (from 1 January as day 1). The data are expressed as mean date ± standard deviation (SD). Data from tree and nut measurements are also expressed as means ± standard deviation (SD). The statistical analysis of all these data was performed with the Statistical Package for the Social Sciences (2011) [29].

## 5. Conclusions

“Leto” is a walnut cultivar of great interest, due to the combination of its characteristics, i.e., small tree size, high lateral fruiting, mid-early vegetation season and excellent fruit characteristics, comparable to those of one of the top walnut cultivars “Chandler”. The main advantage of this cultivar is the small size of the tree, which allows easier harvesting, for example, in cases of small available areas (2–8 acres), and in semi-mountainous and disadvantaged areas, where there is no possibility of mechanization of harvesting. The “Leto” cultivar enables significantly denser plantings per acre compared to its competitor “Chandler”. It is suitable as the main cultivar, where the last spring frost occurs in late March to early April, making it a promising cultivar not only for Greece but also for other regions with similar climatic conditions.

## Figures and Tables

**Table 1:** Growth characteristics, inflorescence and fruiting habit of “Leto” walnut cultivar compared to its parental cultivars “Gustine” and “Pedro” and the reference cultivar “Chandler”, evaluated from 2010 to 2019.

Characteristics ^1, 2, 3^	“Leto”	“Gustine”	“Pedro”	“Chandler”
Tree vigor	Low (3)	Low (3)	Low (3)	Intermediate (5)
Growth habit	Spreading (3)	Spreading (3)	Spreading (3)	Semi-erect (2)
Branching	Dense (7)	Dense (7)	Dense (7)	Dense (7)
Dichogamy	Protandry	Protandry	Protandry	Protandry
Lateral bud flowering (%)	90	90	80	90
First catkin-bearing year	3rd	3rd	3rd	4th
Flowering precocity (years to first female flower)	1st	1st	1st	1st
Catkin abundance	Intermediate (5)	Intermediate (5)	Intermediate (5)	Intermediate (5)
Hull dehiscence	Dehiscent (3)	Slightly dehiscent (2)	Dehiscent (3)	Dehiscent (3)

^1^ According to IPGR (1994) descriptors for walnut. ^2^ Numbers in parentheses given refer to the respective description according to IPGR (1994). ^3^ Observation from ten (10) trees per cultivar.

**Table 2:** Average tree yield (years 2010–2019) and dimensions (years 2018–2019) of “Leto” walnut cultivar compared to its parental cultivars “Gustine” and “Pedro” and the reference cultivar “Chandler”.

Characteristics ^1, 2, 3^	“Leto”	“Gustine”	“Pedro”	“Chandler”	*p*-Value ^4^
Mean tree yield (in-shell walnut—kg)	29.37 ± 4.05	30.26 ± 4.78	28.01 ± 5.21	34.56 ± 6.34	0.345
Tree crown radius (m)	3.78 ± 0.06 ^a^	4.35 ± 0.39 ^b^	4.15 ± 0.22 ^ab^	4.47 ± 0.34 ^b^	0.031
Tree trunk circumference (m)	0.89 ± 0.02 ^a^	1.17 ± 0.08 ^b^	1.13 ± 0.20 ^b^	1.17 ± 0.08 ^b^	0.044

^1^ According to IPGR (1994) descriptors for walnut. ^2^ Numbers in parentheses given refer to the respective description according to IPGR (1994). ^3^ Measurements from ten (10) trees per cultivar. ^4^ *p*-value: probability values. Data are presented as means ± SD; different superscripts indicate significant differences between means.

**Table 3:** Average phenological characteristics of “Leto” walnut cultivar compared to its parental cultivars “Gustine” and “Pedro” and the reference cultivar “Chandler”, evaluated from 2010 to 2019.

Characteristics ^1, 2, 3^	“Leto”	“Gustine”	“Pedro”	“Chandler”	*p*-Value ^4^
Date of bud break	26 March ± 6.61 days ^a^	23 March ± 6.00 days ^a^	31 March ± 5.16 days ^b^	6 April ±8.03 days ^c^	0.001
First female bloom date	11 April ± 5.84 days ^a^	6 April ± 8.29 days ^b^	13 April ± 5.42 days ^a^	27 April ± 6.13 days ^c^	0.001
Last female bloom date	22 April ± 6.11 days ^a^	18 April ± 8.08 days ^b^	25 April ± 5.93 days ^a^	9 May ± 6.31 days ^c^	0.001
First male bloom date	3 April ± 6.57 days ^a^	31 March ± 6.36 days ^a^	8 April ± 5.09 days ^b^	18 April ± 6.26 days ^c^	0.001
Last male bloom date	16 April ± 5.95 days ^a^	13 April ± 7.26 days ^a^	20 April ± 5.51 days ^b^	29 April ± 5.81 days ^c^	0.001
Male and female overlap blooming (%)	57.56 ± 12.88 ^a^	57.83 ± 11.67 ^a^	60.46 ± 11.23 ^a^	24.31 ± 12.33 ^b^	0.001
Harvest date	22 Sept. ± 3.10 days ^a^	19 Sept. ± 3.47 days ^b^	23 Sept. ± 3.00 days ^a^	2 Octob. ± 6.29 days ^c^	0.001

^1^ According to IPGR (1994) descriptors for walnut. ^2^ Numbers in parentheses given refer to the respective description according to IPGR (1994). ^3^ Measurements from ten (10) trees per cultivar. ^4^ *p*-value: probability values. Data are presented as means ± SD; Different superscripts indicate significant differences between means.

**Table 4:** Susceptibility to biotic and abiotic factors of “Leto” walnut cultivar compared to its parental cultivars “Gustine” and “Pedro” and the reference cultivar “Chandler”, assessed for two years (2018–2019).

Characteristics ^1, 2^	“Leto”	“Gustine”	“Pedro”	“Chandler”
Sunburn susceptibility of hull ^3^	Intermediate (5)	High (7)	High (7)	Low (3)
Susceptibility to codling moth (*Cydia pomonella* L.) ^4^	Intermediate (5)	Intermediate (5)	Intermediate (5)	Intermediate (5)
Susceptibility to walnut blight (*Χanthomonas arboricola * pv. *juglandis*) ^4^	Intermediate (5)	Very high (9)	Intermediate (5)	Low (3)

^1^ According to IPGR (1994) descriptors for walnut. ^2^ Numbers in parentheses given refer to the respective description according to IPGR (1994). ^3^ Observation from ten (10) trees per cultivar. ^4^ Observation from one hundred (100) nuts per cultivar.

**Table 5:** Νut and kernel traits of “Leto” walnut cultivar compared to its parental cultivars “Gustine” and “Pedro” and the reference cultivar “Chandler”, assessed from 2010 to 2019.

Characteristics ^3^	“Leto”	“Gustine”	“Pedro”	“Chandler”
Nut size ^1^	Medium (5)	Medium (5)	Medium (5)	Medium (5)
Nut: shape in longitudinal section through suture ^1^	Ovate (4)	Ovate (4)	Ovate (4)	Ovate (4)
Nut: shape in longitudinal section perpendicular to suture ^1^	Trapezium (6)	Ovate (4)	Broad trapezium (5)	Ovate (4)
Nut: shape in cross section ^1^	Oblate (1)	Circular (2)	Oblate (1)	Circular (2)
Nut: shape of base perpendicular to suture ^1^	Rounded (2)	Rounded (2)	Rounded (2)	Rounded (2)
Nut: shape of apex perpendicular to suture ^1^	Rounded (2)	Pointed (1)	Truncate (3)	Rounded (2)
Nut: prominence of apical tip ^1^	Medium (5)	Medium (5)	Medium (5)	Weak (3)
Nut: position of pad on suture ^1^	On upper 2/3 of nut (2)	On upper 2/3 of nut (2)	On upper 2/3 of nut (2)	On upper half of nut (1)
Nut: prominence of pad on suture ^1^	Medium (5)	Medium (5)	Medium (5)	Medium (5)
Nut: width of pad on suture ^1^	Narrow (3)	Narrow (3)	Broad (7)	Narrow (3)
Nut: depth of groove along pad on suture ^1^	Medium (5)	Medium (5)	Deep (7)	Shallow (3)
Nut: structure of surface of shell ^1^	Slightly grooved (1)	Slightly grooved (1)	Embossed (4)	Slightly grooved (1)
Nut: thickness of shell ^1^	Thin (3)	Thin (3)	Very thin (1)	Very thin (1)
Nut: adherence of two halves of shell ^1^	Medium (5)	Medium (5)	Very weak (1)	Weak (3)
Nut: thickness of primary and secondary membranes ^1^	Thin (3)	Thin (3)	Medium (5)	Thin (3)
Kernel color ^1^	Light (3)	Light (3)	Medium (5)	Very light (1)
Ease of removal of kernel halves ^1^	Easy (3)	Easy (3)	Very Easy (1)	Very Easy (1)
Nut shape ^2^	Ovate (4)	Ovate (4)	Short trapezoid (5)	Ovate (4)
Shell texture ^2^	Medium (5)	Medium (5)	Rough (7)	Smooth (3)
Shell color ^2^	Light (3)	Light (3)	Medium (5)	Medium (5)
Shell integrity ^2^	Complete shell (3)	Complete shell (3)	Intermediate (2)	Complete shell (3)
Kernel fill ^2^	Well (7)	Well (7)	Well (7)	Well (7)

^1^ According to UPOV (TG/125/6) (1999) descriptors for walnut; numbers in parentheses given refer to the respective description. ^2^ According to IPGR (1994) descriptors for walnut; numbers in parentheses given refer to the respective description. ^3^ Observation from twenty (20) nuts per cultivar.

**Table 6:** Average nut and kernel measurements of “Leto” walnut cultivar compared to its parental cultivars “Gustine” and “Pedro” and the reference cultivar “Chandler”, evaluated from 2010 to 2019.

Characteristics ^3^	“Leto”	“Gustine”	“Pedro”	“Chandler”	*p*-Value ^4^
Nut height (mm) ^1^	40.57 ± 1.63 ^a^	41.03 ± 1.85 ^ac^	38.36 ± 1.25 ^b^	41.94 ± 1.80 ^c^	0.001
Nut width (mm) ^1^	31.92 ± 1.91 ^a^	32.29 ± 2.49 ^ab^	34.04 ± 1.32 ^c^	33.20 ± 1.42 ^bc^	0.002
Nut thickness (mm) ^1^	31.45 ± 1.04 ^a^	31.20 ± 0.80 ^a^	36.36 ± 1.37 ^c^	35.44 ± 0.93 ^b^	0.001
Nut roundness ^1^	1285.55 ± 77.44 ^a^	1302.83 ± 87.10 ^ab^	1351.01 ± 79.23 ^b^	1439.92 ± 83.25 ^c^	0.001
Shell thickness (mm) ^2^	2.10 ± 0.17 ^a^	2.16 ± 0.21 ^a^	2.05 ± 0.21 ^a^	1.91 ± 0.29 ^b^	0.008
In shell nut weight (g) ^2^	12.96 ± 0.61 ^ab^	13.39 ± 0.76 ^bc^	12.67 ± 0.83 ^a^	13.68 ± 1.25 ^c^	0.002
Kernel weight (g) ^2^	6.40 ± 0.54 ^ab^	6.47 ± 0.54 ^ab^	6.06 ± 0.67 ^a^	6.81 ± 0.73 ^b^	0.004
Kernel percentage (%) ^2^	49.37 ± 2.81 ^bc^	48.28 ± 2.39 ^ab^	47.85 ± 1.58 ^a^	49.78 ± 1.88 ^c^	0.020

^1^ According to UPOV (TG/125/6) (1999) descriptors for walnut; numbers in parentheses given refer to the respective description. ^2^ According to IPGR (1994) descriptors for walnut; numbers in parentheses given refer to the respective description. ^3^ Measurements from twenty nuts (20) evaluated per cultivar. ^4^ *p*-value: probability values. Data are presented as means ± SD; Different superscripts indicate significant differences between means.

## Data Availability

All research data were presented in this contribution.
